# Supplementary figures and images for: KANSL3 directs transcriptional programs essential for hepatic metabolism and differentiation
Source: Life Sci Alliance. 2025 Oct 3;8(12):e202503238. doi: 10.26508/lsa.202503238 (PMC12495240; doi:10.26508/lsa.202503238)

Uncropped Western blots for Figure 1B

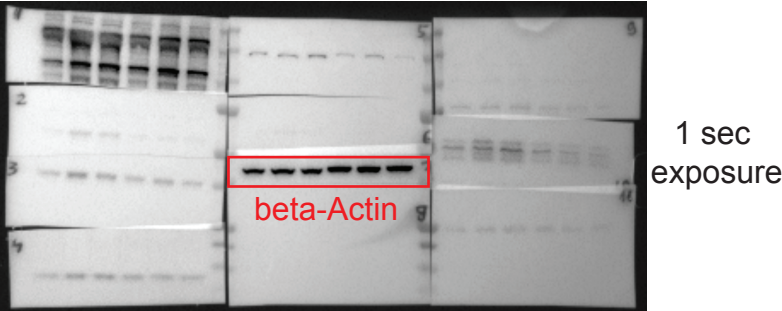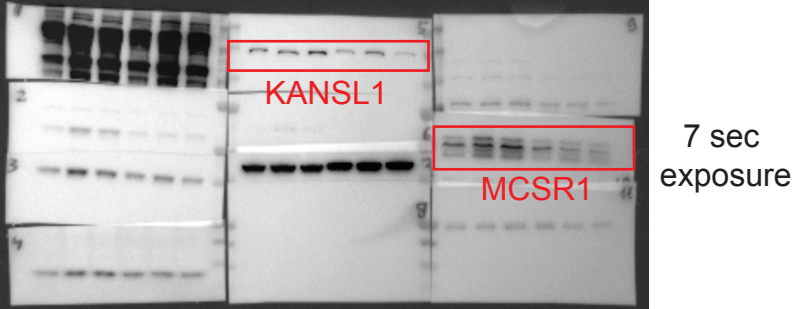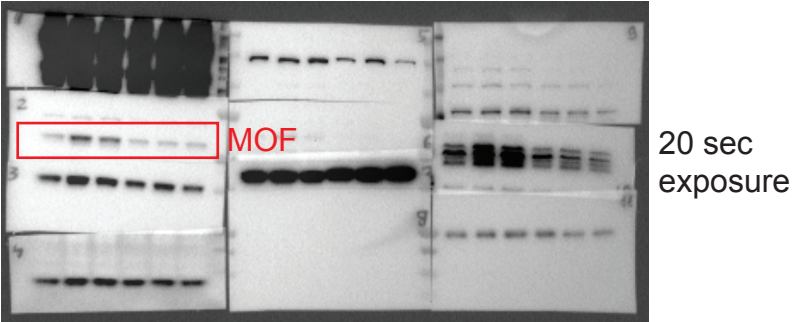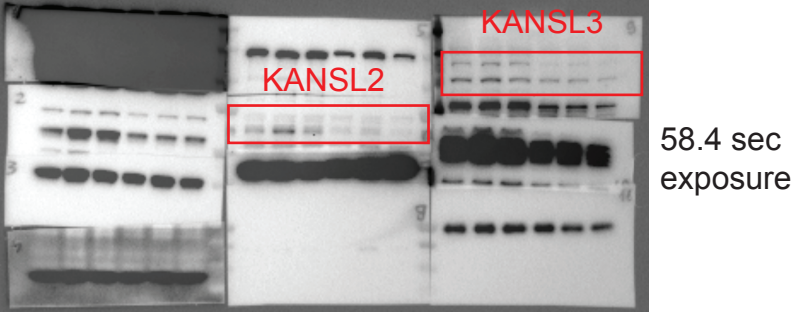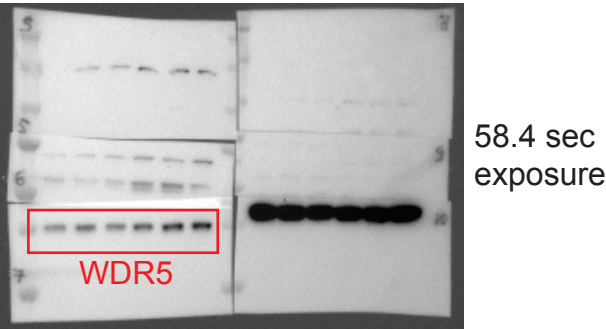

Supplement: Supplementary file 1 [file LSA-2025-03238_SdataF1.pdf]

Uncropped Western blots for Supplemental Fig. S7E

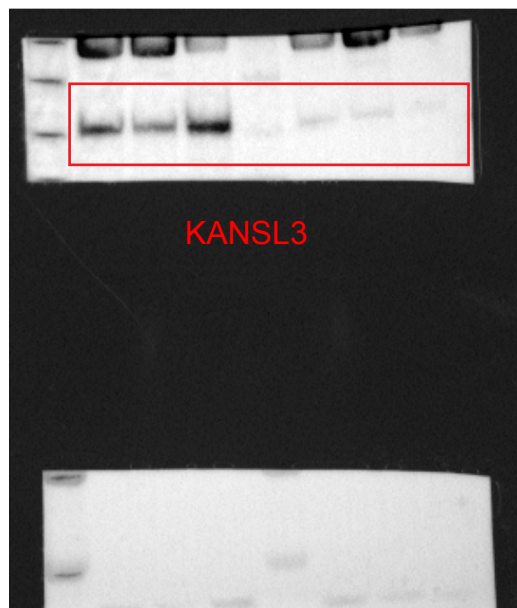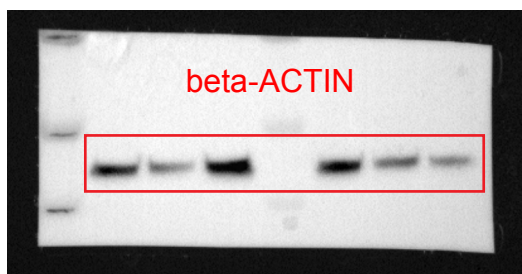

Supplement: Supplementary file 7 [file LSA-2025-03238_SdataFS7.pdf]
